# Detection of emergency department patients at risk of dementia through artificial intelligence

**DOI:** 10.1002/alz.70334

**Published:** 2025-06-02

**Authors:** Inessa Cohen, Richard Andrew Taylor, Haipeng Xue, Isaac V. Faustino, Natalia Festa, Cynthia Brandt, Emily Gao, Ling Han, Siddarth Khasnavis, James M. Lai, Adam P. Mecca, Atharva Vinay Sapre, Juan Young, Michael Zanchelli, Ula Hwang

**Affiliations:** ^1^ Department of Emergency Medicine Yale School of Medicine New Haven Connecticut USA; ^2^ Department of Biomedical Informatics and Data Science Yale University School of Medicine New Haven Connecticut USA; ^3^ Program of Computational Biology and Bioinformatics Yale University School of Medicine New Haven Connecticut USA; ^4^ Department of Internal Medicine Yale School of Medicine New Haven Connecticut USA; ^5^ Department of Psychiatry Yale School of Medicine New Haven Connecticut USA; ^6^ Division of Geriatric Medicine and Palliative Care Department of Internal Medicine New York University Grossman School of Medicine New York New York USA; ^7^ Geriatric Research Education and Clinical Center James J. Peters VAMC Bronx New York USA; ^8^ Departments of Emergency Medicine and Population Health New York University Grossman School of Medicine New York New York USA

**Keywords:** care transition intervention, cognitive impairment, dementia, early dementia detection, emergency department

## Abstract

**INTRODUCTION:**

The study aimed to develop and validate the Emergency Department Dementia Algorithm (EDDA) to detect dementia among older adults (65+) and support clinical decision‐making in the emergency department (ED).

**METHODS:**

In a multisite retrospective study of 759,665 ED visits, electronic health record data from Yale New Haven Health (2014–2022) were used to train three supervised and semi‐unsupervised positive‐unlabeled machine learning models (XGBoost, Random Forest, LASSO). A separate test set of 400 ED encounters underwent adjudicated chart review for validation.

**RESULTS:**

EDDA achieved an area under the receiver‐operating characteristic curve (AUROC) of 0.85 in the test set and 0.93 in the validation set. Positive‐unlabeled learning improved performance. Agreement between EDDA and clinician‐adjudicated dementia diagnoses was moderate (kappa = 0.50), with 17% of EDDA‐positive patients having undiagnosed probable dementia.

**DISCUSSION:**

EDDA enhances dementia detection in the ED, with potential for real‐time implementation to improve patient outcomes and care transitions.

**Highlights:**

- Developed a machine learning algorithm using electronic health record data to detect dementia in the emergency department (ED).
- Algorithm designed to balance detection accuracy with ease of ED implementation.
- Parsimonious model with limited but predictive variables selected for rapid ED use.
- Focused on real‐time application, optimizing ED workflows, and clinician support.
- Aims to enhance ED dementia detection, patient safety, and care coordination.

## 1 BACKGROUND

Increasing population age, coupled with greater prevalence of multimorbidity,^1^, ^2^ has led to a surge in emergency department (ED) visits by older adults.^3^, ^4^ Persons living with dementia (PLWD) are twice as likely to use the ED^5^, ^6^ and 1.5 times more likely to have avoidable visits.^7^, ^8^ Those diagnosed with dementia have been found to have a surge in ED visits in the months prior to their formal diagnoses, indicating an opportunity for earlier recognition and intervention,^9^, ^10^ especially since PLWD seen in the ED face higher risks of poor outcomes and are more likely to return.^5^, ^6^ Despite the importance of dementia recognition to informed medical decision‐making and care coordination, it is often under‐recognized in the ED,^11^, ^12^, ^13^, ^14^ especially in underrepresented populations.^15^ Although screening for cognitive impairment is recommended,^13^, ^16^, ^17^ the often chaotic environment of the ED, provision of care under the pressure of significant multitasking, and balancing of acute and critical care, limit the ability of emergency clinicians to appropriately assess for common geriatric syndromes.^18^, ^19^ This under‐recognition occurs in two distinct subgroups: older adults with already established dementia diagnoses that are not readily apparent due to incomplete or variable documentation in the electronic health record (EHR) or missed by busy emergency clinicians and older adults with undiagnosed or subclinical dementia whose cognitive impairment is not yet formally identified. This under‐recognition of cognitive impairment and dementia can exacerbate safety risks, such as poor comprehension of discharge instructions, or perpetuate missed diagnoses during inpatient care, resulting in longer hospital stays, accelerated functional and cognitive decline, and increased health care costs.^20^, ^21^, ^22^, ^23^, ^24^, ^25^ Consensus by patients, care partners, and transdisciplinary geriatric emergency medicine clinicians has made detection of dementia in the ED a priority for emergency care.^26^

Experts suggest that next‐generation clinical pathways for Alzheimer's care must embrace a paradigm shift toward enhanced detection and comprehensive dementia care.^27^ This shift requires moving from the current model of late‐stage diagnosis to one that employs digitally driven decision‐making algorithms for risk stratification, early detection, and timely intervention. The widespread adoption of EHRs and advances in computing capacity now allow for the use of machine learning techniques and EHR‐integrated clinical decision support, opening new possibilities for predictive and preventive care. In recent years, a variety of dementia detection tools and algorithms have been developed, primarily for outpatient or inpatient settings.^28^, ^29^, ^30^, ^31^, ^32^, ^33^, ^34^ However, few are designed for real‐time application, and have been tailored specifically for the ED, where rapid decision‐making is essential and timely recognition of cognitive impairment could benefit both diagnosed and undiagnosed patients with dementia.

The objective of this study was to develop a machine learning algorithm, the Emergency Department Dementia Algorithm (EDDA), designed specifically to improve the detection of dementia of older adults in the ED setting. In particular, EDDA aims to address two key gaps in dementia recognition: (1) identify patients who do not yet have two or more historical International Classification of Diseases (ICD)–based dementia diagnoses, (2) consolidate disparate EHR data to increase confidence in known diagnoses, and (3) offer different thresholds of detection of dementia presence that can be adapted for real‐time clinical use. With future implementation applicability as a core focus, we prioritized a parsimonious model that balances accuracy with simplicity, ensuring that it is both feasible for real‐time application and minimally disruptive to clinicians’ workflows. By improving dementia detection across these two subgroups, EDDA is intended to support informed decision‐making, safer disposition planning, and timely referrals for comprehensive evaluation and coordinated care.

## 2 METHODS

### 2.1 Study design

This was a retrospective observational study of ED visits from patients 65+ years of age, who presented to one of nine hospital EDs in a regional hospital network in the Northeast United States between January 1, 2014 and March 31, 2022. Hospitals included two nonacademic urban, two academic urban, and five nonacademic suburban sites. Data included individuals from underrepresented and minoritized racial and ethnic groups, as well as those with functional impairments. Our institutional review board approved this study and waived the need for informed consent (Human Investigation Committee#: 2000033102). This study was performed in accordance with the ethical standards as laid down in the 1964 Declaration of Helsinki.

### 2.2 Data sources

Structured EHR data were extracted from the institutional database on the Yale New Haven Health computational health platform. Data pulled included visit‐level features from different domains, including patient demographics, past medical history (historical diagnoses), vitals, prescription medications, laboratory measurements, procedures, and prior encounters.

**FIGURE 1:**
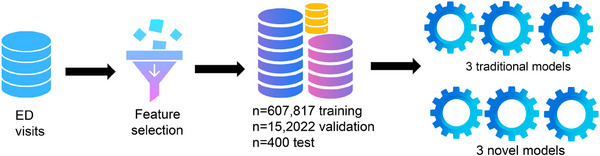
Machine learning pipeline. Emergency department visits underwent feature selection, followed by data partitioning into a training, test, and validation sets. We evaluated three traditional supervised machine learning models and three novel semi‐supervised models. ED, emergency department.

EHR data included were initially split into a *training se*t (80%) and a cross‐*validation set* (20%) (Figure 1). A separate clinician expertise adjudicated (CEA) *test set* of 400 randomly selected ED encounters was reserved, with these cases reviewed by geriatric psychiatrists and geriatricians to adjudicate the presence or absence of dementia based on chart review of EHRs.

### 2.3 Outcomes

*Training and Validation Set*—For the training and cross‐validation sets, dementia was defined as the presence or absence of two or more historical dementia ICD‐9 or ICD‐10 diagnosis codes in any period of time before an index ED visit. Qualifying dementia diagnoses followed Bynum definition standards.^35^

**RESEARCH IN CONTEXT**

1. **Systematic review**: A systematic review was conducted by searching major databases (PubMed, Embase, Cochrane Library) for studies focused on dementia detection in acute care settings, particularly the emergency department (ED). Inclusion criteria were limited to studies implementing machine learning or clinical decision support tools. Studies were evaluated for sample size, methodology, and real‐time applicability, highlighting significant gaps in the literature for ED‐specific tools and real‐time dementia detection.
2. **Interpretation**: This study provides a novel machine learning algorithm enabling rapid, in situ use in the ED. Unlike existing outpatient or inpatient models, our algorithm addresses ED‐specific needs, contributing to improved recognition of dementia, and facilitating timely care coordination.
3. **Future directions**: Future research should validate this algorithm in diverse ED environments, refining predictors for accuracy and examining its impact on clinical outcomes and ED workflow efficiency. Further work is needed to integrate the model within electronic health records for seamless, real‐time use.

*Reference Standard Clinician Expertise Adjudication (CEA) Test Set*—A random subsample of 400 unique patient encounters was used to construct a cohort for comprehensive EHR review and adjudication of cognitive status by clinician‐reviewers with domain expertise (geriatrics and geriatric psychiatry), in contrast to the outcome of presence or absence of ICD‐based dementia. As prior studies have cautioned about the use of claims‐based dementia ascertainment and its low sensitivity,^36^, ^37^ we created a clinician‐expertise adjudicated test set. The clinician reviewers modified an existing protocol for the classification of cognitive status using EHR data, led by a senior geriatric psychiatrist.^38^ The standards and criteria for this Modified Dementia Adjudication Protocol (MDAP) are summarized in Appendix SA1. They reviewed practice cases until achieving high interrater agreement (Fleiss kappa > 0.75) on a randomly selected subset of practice encounters. After meeting the predetermined standards for agreement on practice cases, the clinician‐reviewers were each randomly assigned 100 patient encounters for comprehensive EHR review. For each patient, each clinician‐reviewer thoroughly reviewed the EHR for clinically relevant information from the index ED encounter date and earlier. They used available information to inform their classification of cognitive impairment, its stage (mild cognitive impairment [MCI] or dementia), and, when applicable, the severity of dementia. They also rated their level of certainty for each classification, as outlined in the MDAP.^38^ Each assessment was informed by the *Diagnostic and Statistical Manual of Mental Disorders, Fifth Edition* (DSM‐5) and National Institute on Aging–Alzheimer's Association (NIA‐AA)_ diagnostic guidelines.^39^, ^40^, ^41^

### 2.4 Feature engineering

The datasets were augmented with additional features that included important visit‐level summary statistics. These features were based on a literature review of risk factors for dementia, contextual knowledge, and extensions of previous work.^42^ This included features such as the number of prior ED visits in the past 6 months, prior outpatient visits, and comorbidity scores such as the Charlson comorbidity index and Elixhauser. Features included only structured data available at the the time of the ED visit. Missing values were imputed with the median, zero, and “missing” categories for continuous (e.g., lab values and age), discrete (e.g., number of magnetic resonance imaging [MRI] studies and ED visits), or categorical features (e.g., sex, race, and ethnicity), respectively. Features that had ≥99% missing values (e.g., rare lab values) were dropped. Discrete features were discretized. Continuous features were normalized, and any outliers were winsorized at the 99th percentile. Categorical features with rare labels were collapsed and one‐hot encoded. Medications and comorbidities were grouped using the Anatomical Therapeutic Chemical (ATC) Classification System^43^ and Clinical Classification Software categories.^44^ Features that were highly correlated (*r* = 0.9) or redundant (tolerance of 0.99) were dropped. Feature preprocessing and engineering were performed using Python 3.8.19 using the Feature‐engine package.^45^

### 2.5 Model development

To address potential issues including overfitting, collinearity, and sparse data bias, three machine learning models were employed: XGBoost,^46^ Random Forest,^47^ and least absolute shrinkage and selection operator (LASSO),^48^ with dementia as the binary primary outcome (yes/no) Each machine learning model was implemented in two separate pipelines: traditional supervised machine learning and semi‐supervised positive‐unlabeled learning.^49^

In the positive‐unlabeled learning scenario, a subset of 20,000 true dementia‐positive cases—identified via ICD codes^35^–were embedded deliberately within the unlabeled class. This approach allowed the models to discover hidden dementia cases within the unlabeled data, thereby simulating real‐world conditions where complete labeling is unavailable. Each model underwent comprehensive hyperparameter tuning with 5‐fold cross‐validation to ensure optimal model performance and generalizability to unseen data. For the Random Forest model, key parameters included the number of trees, maximum tree depth, and the minimum samples per split. For XGBoost, tuning focused on the learning rate, maximum tree depth, and regularization terms. The LASSO model involved optimizing the regularization strength and tolerance. All hyperparameter optimization was performed using the Tree‐structured Parzen Estimator algorithm, selecting the best parameters by minimizing cross‐validation error as measured by the area under the receiver‐operating characteristic curve (AUROC) and the area under the precision‐recall curve (AUPR). Final model performance was validated against a reference‐standard test set of 400 unique person encounters with clinician‐adjudicated dementia status, ensuring the robustness and clinical relevance of the predictive models.

### 2.6 Performance evaluation

After hyperparameter tuning, model performance was evaluated on both the validation and external test datasets to assess their generalization capabilities. Feature selection was conducted using recursive feature elimination to identify the top 20, 50, 100, and 200 predictive features. Each subset of features was evaluated across the Random Forest, XGBoost, and LASSO models, with comparisons based on area under the curve receiver‐operating characteristic and AUCPR metrics. Ultimately, XGBoost with 50 features was selected as the best‐performing model based on these evaluations.

Model performance was further assessed using metrics such as AUROC, AUPR, recall, and F1 score. Calibration of the models was evaluated using calibration curves, comparing predicted probabilities to actual outcomes. A perfectly calibrated model would align closely with a 45‐degree diagonal line, indicating an ideal correspondence between the predicted and observed outcomes. Calibration performance was visualized by plotting the mean predicted probability against the fraction of positive outcomes across multiple bins.

To ensure that the models perform equitably across different subgroups, we conducted a detailed fairness assessment based on age, sex, race, and ethnic groups. For each subgroup, model performance was evaluated using key metrics, including AUROC, AUPR, and calibration metrics. Calibration was assessed through bootstrapping to estimate confidence intervals (CIs) for intercepts and slopes, allowing us to gauge over‐ or under‐estimation in predicted probabilities. This analysis quantified potential biases in predictions across different groups, with results reported alongside 95% CIs to ensure statistical robustness.

Baseline characteristics of the study sample are presented using frequency and percentage, mean and standard deviation (SD), or median and interquartile range (IQR). Statistical analyses were performed using R version 4.3.2 (R Foundation for Statistical Computing, Austria). Additional analyses, including model development and testing, were conducted in Python version 3.12.5. EDDA performance characteristics are reported for the 400 CEA test set ED encounters, comparing the performance of the EDDA against reference standards of ICD‐based dementia presence versus CEA dementia presence. Sensitivity, specificity, positive predictive value (PPV), negative predictive value (NPV), number needed to screen (NNS), and number needed to treat (NNT) are reported.

## 3 RESULTS

### 3.1 Cohort characteristics

Between January 2014 and March 2022, there were 3,640,261 visits by 1,053,511 patients across Yale New Haven Health. Of those, 760,339 visits (20.9%) were from 213,281 older adults who were 65+ years in age. The median age of the cohort was 77 (IQR: 70.0, 85.0). Overall, most were White (77.8%), female (56.8%), and non‐Latinx (90.3%). The demographic characteristics across training, validation, and test sets were similar (Table 1). Overall, the ICD‐based dementia prevalence was 12.5% (*n* = 26,592) between 2014 and 2022.

**TABLE 1:** Cohort characteristics.

	Training (*N* = 607,817)	Validation (*N* = 152,022)	Test (*N* = 400)	Overall (*N* = 760,239)
Age				
Median (IQR)	77.0 (70.0, 85.0)	77.0 (70.0, 85.0)	77.0 (69.0, 85.0)	77.0 (70.0, 85.0)
Sex				
Female	34,4855 (56.7%)	86,527 (56.9%)	223 (55.8%)	431,605 (56.8%)
Male	262,956 (43.3%)	65,494 (43.1%)	177 (44.3%)	328,627 (43.2%)
Unknown	6 (0.0%)	1 (0.0%)	0 (0%)	7 (0.0%)
Race				
American Indian	1037 (0.2%)	288 (0.2%)	1 (0.3%)	1326 (0.2%)
Asian	6835 (1.1%)	1663 (1.1%)	7 (1.8%)	8505 (1.1%)
Black or African American	76,139 (12.5%)	19,641 (12.9%)	40 (10.0%)	95,820 (12.6%)
Native Hawaiian or Other Pacific Islander	803 (0.1%)	190 (0.1%)	1 (0.3%)	994 (0.1%)
Other	42,360 (7.0%)	11,113 (7.3%)	23 (5.8%)	53,496 (7.0%)
Unknown	7293 (1.2%)	1669 (1.1%)	11 (2.8%)	8973 (1.2%)
White	473,350 (77.9%)	117,458 (77.3%)	317 (79.3%)	591,125 (77.8%)
Ethnicity				
Latinx	52,871 (8.7%)	13,561 (8.9%)	28 (7.0%)	66,460 (8.7%)
Non‐Latinx	548,971 (90.3%)	137,149 (90.2%)	360 (90.0%)	686,480 (90.3%)
Unknown	5975 (1.0%)	1312 (0.9%)	12 (3.0%)	7299 (1.0%)
Language				
English	552,820 (91.0%)	138,002 (90.8%)	362 (90.5%)	691,184 (90.9%)
Other	54,997 (9.0%)	14,020 (9.2%)	38 (9.5%)	69,055 (9.1%)
Insurance				
Medicaid	18,577 (3.1%)	4845 (3.2%)	13 (3.3%)	23,435 (3.1%)
Medicare	530,638 (87.3%)	132,511 (87.2%)	330 (82.5%)	663,479 (87.3%)
Other	11,463 (1.9%)	2835 (1.9%)	10 (2.5%)	14,308 (1.9%)
Private	47,139 (7.8%)	11,831 (7.8%)	47 (11.8%)	59,017 (7.8%)
Charlson score				
Median (IQR)	7.0 (4.0, 10.0)	7.0 (4.0, 10.0)	6.0 (4.0, 9.0)	7.0 (4.0, 10.0)
Elixhauser score				
Median (IQR)	9.0 (0.0, 18.0)	9.0 (0.0, 18.0)	6.0 (0.0, 14.0)	9.0 (0.0, 18.0)

*Note*: Data are *n* (%) unless indicated otherwise. Baseline demographic characteristics for the training, validation and test sets.

Abbreviation: IQR, interquartile range.

### 3.2 Machine learning models

A total of 1641 dementia risk factor features were included from demographics, lab results, and past medical history in the XGBoost, Random Forest, and LASSO models. Ensemble feature selection reduced dimensions from 1641 to 20, 50, 100, and 200 features.[Fig alz70334-fig-0002] The top 20 features in descending order of importance included: geriatric outpatient specialist visits (number), any dementia drug prescriptions (yes/no), minimum mean platelet volume (number), psychotherapeutic drug prescriptions (yes/no), autonomic drug prescriptions (yes/no), prior ED visits in the last 2 years (number), history of schizophrenia/psychotic disorder (yes/no), history of falls (yes/no), history of coma/stupor/brain damage (yes/no), Elixhauser score (number), Charlson score (number), age (number), other procedures ordered in the ED within the past year (number), no previous discharge disposition (yes/no), prior hospitalizations in the past 24 months (number), neurology visits (number), mean corpuscular hemoglobin concentration (number), history of developmental disorders (yes/no), memantine dementia drug prescription (yes/no), donepezil dementia drug prescription (yes/no), and head computerized tomography (CT) ordered in the ED within the past year (number). A SHapley Additive exPlanations plot is included as Figure S1 for a visualization of these top 20 EDDA features highlighting those with highest to lowest predictive impact on the model output.

### 3.3 EDDA performance

**FIGURE 2 alz70334-fig-0002:**
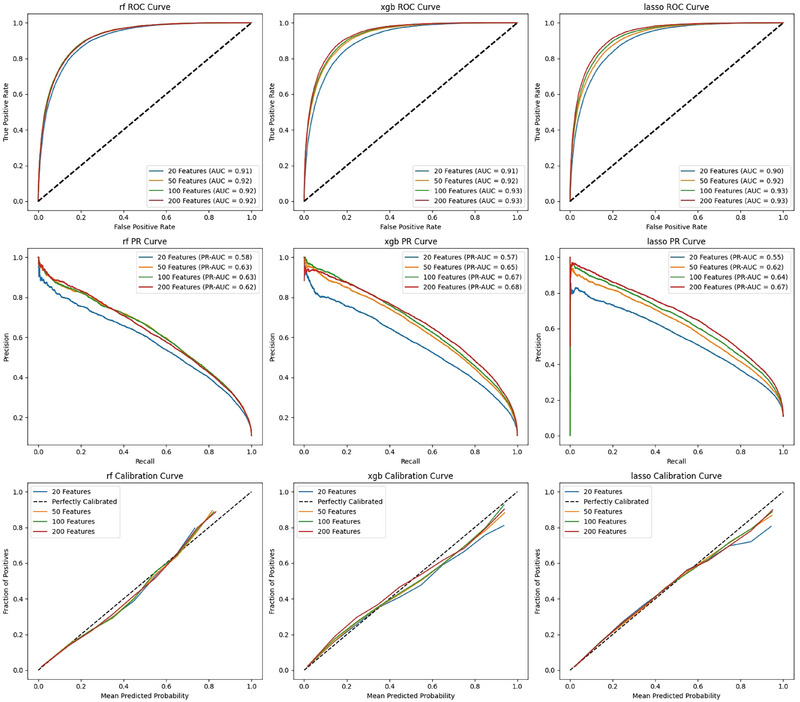
Validation set performance. Machine learning model performance metrics for validation set across three machine learning models. AUCROC, area under the curve receiver‐operating characteristic; AUPR, area under the precision‐recall curve; RF, Random Forest; ROC, receiver‐operating characteristic; XGB, XGBoost.

**FIGURE 3:**
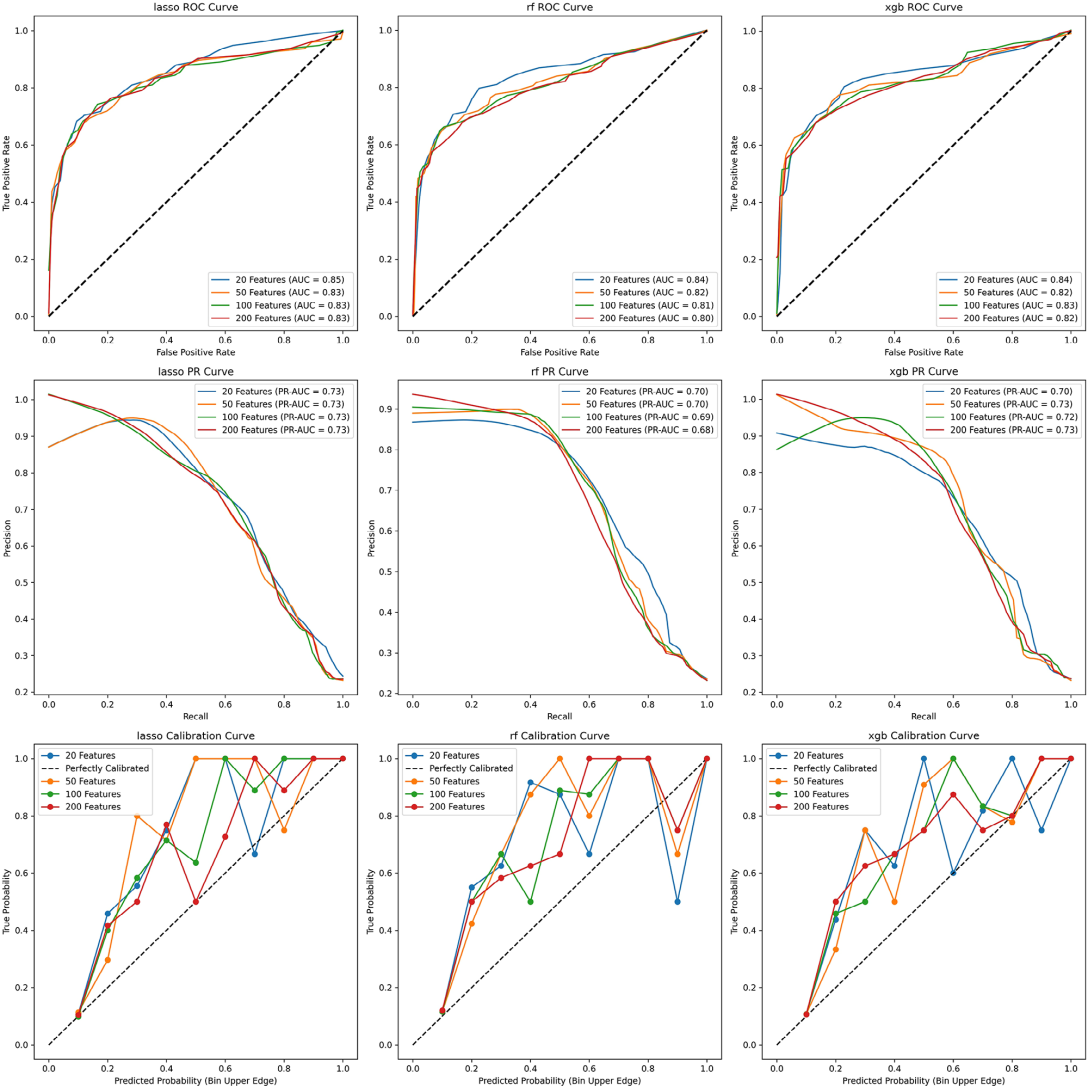
Test set performance. Machine learning model performance metrics for CEA test set. AUCROC, area under the curve receiver‐operating characteristic; CEA, clinician expertise adjudication; RF, Random Forest; ROC, receiver‐operating characteristic; XGB, XGBoost.

Traditional models demonstrated consistent performance across varying features, with AUROCs up to 0.93 in the validation set (Figure 2) and 0.85 in the CEA test set (Figure 3). With positive‐unlabeled learning, the models' ability to recover hidden true positives improved with increasing feature set sizes. The performance incrementally enhanced from 20 to 200 features, with XGBoost outperforming the other models.

### 3.4 Comparison of EDDA detection of the outcome of ICD dementia versus CEA dementia

Using CEA as the gold standard, the EDDA identified dementia with the following results: In 38 known dementia cases (ICD+, CEA+) (ICD true positive), 30 were EDDA positive (66.7%) and 8 were EDDA‐negative (2.5%). For 49 undiagnosed dementia cases (ICD–, CEA+) (ICD false negative), 8 were EDDA positive (17.8%) and 41 were EDDA negative (12.6%). Among four cases with a history of ICD dementia (ICD+, CEA–) (ICD false positive), two were EDDA positive (4.4%) and two were EDDA negative (0.6%). Of 280 cases without dementia (ICD–, CEA–) (ICD true negative), 275 were EDDA negative (84.4%) and five were EDDA positive (11.1%) (Table 2).

**TABLE 2:** Comparison of EDDA against ICD codes and CEA.

Dementia by ICD and CEA	EDDA negative (*N* = 326)	EDDA positive (*N* = 45)	Overall (*N* = 371)
Known dementia (ICD+, CEA+) ICD true positive	8 (2.5%)	30 (66.7%)	38 (9.5%)
Undiagnosed dementia (ICD−, CEA+) ICD false negative	41 (12.6%)	8 (17.8%)	49 (12.3%)
History of ICD dementia (ICD+, CEA−) ICD false positive	2 (0.6%)	2 (4.4%)	4 (1.0%)
No Dementia (ICD−, CEA−) ICD true negative	275 (84.4%)	5 (11.1%)	280 (70.0%)

*Note*: Column percentages are shown. Twenty‐nine patients lacked data for chart review and were excluded. EDDA results are color‐coded: false negative (yellow), true positive (green), true negative (blue), and false positive (red).

Abbreviations: CEA, clinician expertise adjudication; EDDA, Emergency Department Detection Algorithm; ICD, International Classification of Diseases.

### 3.5 EDDA sensitivity, specificity, PPV, NPV, NNT

At the 0.1 threshold, the EDDA model showed the highest sensitivity (0.57), but with lower specificity (0.93) and a PPV (0.72). In contrast, at the 0.9 threshold, the model achieved perfect specificity and PPV, but sensitivity dropped to 0.11, reducing its ability to detect dementia. From a machine learning perspective, the optimal threshold for performance was 0.5, which is at the inflection point on the ROC curve that offers a balance of moderate sensitivity (0.34), high specificity (0.99), high PPV (0.94), and a reasonable NPV (0.83). From a clinical perspective, however, setting the algorithm to perform at a 0.1 threshold may be preferred, as it better captures more individuals at risk of probable dementia, which is critical for a screening tool aimed to improve dementia detection (Table 3).

**TABLE 3:** Predicted dementia probability thresholds.

Threshold	Sensitivity	Specificity	PPV	NPV	NNT	FP	FN	TP	TN
0.1	0.57	0.93	0.72	0.88	1.97	19	37	50	265
0.2	0.53	0.97	0.85	0.87	2.00	8	41	46	276
0.3	0.44	0.98	0.84	0.85	2.43	7	49	38	277
0.4	0.41	0.99	0.92	0.85	2.48	3	51	36	281
0.5	0.34	0.99	0.94	0.83	2.96	2	57	30	282
0.6	0.29	0.99	0.93	0.82	3.57	2	62	25	282
0.7	0.22	1.00	0.95	0.81	4.65	1	68	19	283
0.8	0.16	1.00	1.00	0.80	6.21	0	73	14	284
0.9	0.11	1.00	1.00	0.79	8.70	0	77	10	284

*Note*: Predicted probability of dementia disease thresholds for and corresponding numbers of false positives, true positives, false negatives, and true negatives and values for sensitivity, specificity, PPV and NPV, and NNT.

Abbreviations: FN, false negative; FP, false positive; ICD, International Classification of Diseases; NNT, number‐needed‐to‐treat; NPV, negative predictive value; PPV, positive predictive value; TN, true negative; TP, true positive.

## 4 DISCUSSION

In our test sample of older ED patients randomly selected and reviewed by clinician experts, the ED Dementia Algorithm was able to identify 10.2% (30 of 371) of ED patients at the time of an ED encounter who had probable dementia, including both patients with diagnosed and undiagnosed dementia. The EDDA had high performance, with AUROCs as high as 0.93 and 0.85 in validation and test datasets, and AUPRs as high as 0.68 and 0.73, respectively. Twenty‐three percent (87 of 371) of the test set was identified by clinician experts (geriatric psychiatrists, psychiatrists, and geriatricians) as having probable dementia, and 12.1% (45 of 371) identified by EDDA with probable dementia risk. Of these 45 EDDA‐positive patients, 66.7% (30) had known ICD dementia, and 17.8% (8) had no prior ICD dementia codes. Thus, one of five EDDA‐positive patients may have undiagnosed probable dementia patients. The algorithm can serve as a digital automated tool, requiring only as little as 20 structured EHR data features to help busy ED clinicians better recognize patients at risk of cognitive impairment—both those with diagnosed and undiagnosed conditions. Our findings support the potential of machine learning in enhancing dementia detection in the ED, with XGBoost showing the best performance. Although traditional model methods are robust, incorporating positive‐unlabeled learning enhances the models' predictive power by effectively identifying hidden dementia cases and reducing the need for manual chart review, saving valuable time and resources. The integration of these advanced methods as screening tools into clinical workflows can direct and focus busy, multitasking clinicians to complete brief cognitive assessments with screening tools that can direct clinicians to improve early dementia identification, enabling more timely and targeted patient care. Furthermore, these methods have been optimized for integration into real‐time industry platforms, ensuring the applicability of EDDA in dynamic, operational environments. Future studies should include prospective validation and assessment of implementation impact on clinical care.

The ED is an untapped health care setting to expand targeted efforts to improve dementia detection in some of the most vulnerable and socioeconomically challenged populations. It is one of the few health care settings where patients of all backgrounds and conditions are seen and cared for, regardless of demography, socioeconomic factors, or conditions.^50^ Recent studies demonstrate that ED offers a critical, but time‐limited opportunity to alter health trajectories and catch missed conditions like dementia for vulnerable patients, with surges in ED visits in the months prior to formal diagnoses.^9^, ^10^ The ED is already undergoing transformation to better address the special care needs of older patients.^51^ By capitalizing on this momentum, it has become a place for detecting geriatric syndromes, including undiagnosed conditions like dementia.^40^ This proactive approach could facilitate referrals, improve access to care, ultimately reducing preventable ED revisits and hospitalizations in the future and increasing research opportunities, especially with the advent of advanced biomarker detection tools and imaging modalities. Because dementia is such a complex disease to diagnose and is frequently unrecognized in multiple settings, use of artificial intelligence (AI)–driven detection, like the EDDA, can improve clinician recognition in time‐ and resource‐constrained settings, such as the ED. For individuals with diagnosed disease, clinicians and members of the care team can use this information as reminders to strengthen and reinforce medical decision‐making and care coordination, including safe disposition planning. For individuals with previously undiagnosed disease, ED physicians have a unique opportunity to direct these individuals into appropriate diagnostic and care coordination pathways.

Providing clinicians with a clearer sense of underlying dementia can aid clinicians in evaluating and risk‐stratifying these presentations, while encouraging longitudinal providers to develop appropriate care plans to reduce avoidable recurrent presentations. Real‐time dementia risk stratification plays a crucial role in improving the care of PLWD in the ED. The ED is often a critical point of care for older adults, and identifying and recognizing the presence of dementia in real time can significantly impact patient outcomes and health care resource utilization. PLWD are more than twice as likely to be hospitalized, compared to those in the same age group without Alzheimer's disease (AD) and related dementias (ADRD).^52^ Meanwhile, early detection, as part of emergency care, could help with a reduction in readmission, unnecessary transfers, and the overall time in the hospital (door‐to‐discharge time), promoting improved outcomes in patient care.^53^ When ED physicians are unaware of a dementia diagnosis, this can lead to poor quality of care, measured by delayed and missed diagnoses and increased patient morbidity.^53^

Detection of ADRD in the ED setting also plays a vital role in the health and well‐being of patients, their families, and society. Recognition of dementia in those with a known dementia is critical as part of emergency care for older ED patients. Benefits those with probable or even already diagnosed dementia include establishing (1) more pathways and supports to coordinate complex medical decision making both with and for the patient and the support of surrogate decision makers, (2) the ability to institute well‐established and straightforward preventive measures for geriatric syndromes that PLWD are at increased risk for developing, like delirium, and (3) the ability to make informed decisions regarding safe discharge or hospitalization—individuals with dementia with appropriate social supports ideally could be safely discharged home with home hospital services or to inpatient with age‐friendly supports.

Thus, the EDDA is a novel tool that can enable the identification and detection of older ED patients at risk of probable undiagnosed disease. Benefits here include (1) the ability to refer patients from the ED for formal diagnosis and management, which can facilitate intervening upon modifiable risk factors for disease progression. With new technologies, (2) the ability to refer to outpatient settings that can provide patients and family members with the necessary home‐ and community‐based supports to facilitate safely aging in place (including support with driving safety assessments, fall‐prevention, and medication management). Use of the EDDA provides (3) evidence of feasibility with such technologies and tools. Even a modest detection rate can be valuable in clinical practice, where recognizing even a fraction of previously unrecognized cases may prompt timely evaluation and referral. Finally, the EDDA allows (4) the ability to be flexible with threshold customizations. The sensitivity reported in this study is based on a specific probability threshold. Lowering the threshold could potentially improve sensitivity (i.e., flag more individuals), although this would increase false positives for dementia risk.

### 4.1 Limitations

This study has several limitations that should be acknowledged. First, the retrospective nature of the study may introduce biases related to data quality and completeness. The data extracted from the EHR may have been incomplete or contain inaccuracies, which could affect model performance. Although the diversity of the sample is determined by the available EHR data, further research may be necessary to address any disparities in representation, particularly among certain marginalized groups.

In addition, the use of ICD codes as part of the reference standard for dementia diagnosis has limitations, as these codes may not fully capture all cases of dementia, leading to potential misclassification.^36^, ^37^, ^54^ This was our study rationale for a CEA test set that provides a more robust reference than ICD‐based dementia ascertainment alone. The CEA process using clinician expert retrospective review of records nonetheless may still underestimate truly “silent” dementia cases. The moderate agreement between EDDA and clinician‐adjudicated diagnoses suggests room for improvement, particularly in optimizing the model to align more closely with clinical evaluations. Although the EDDA had specificity as high as 0.93 when identifying individuals with ICD‐based dementia, its sensitivity was only considered modest in performance when set at the lowest threshold for detection. As a screening tool to rule out the risk of probable dementia, it would ideally have higher sensitivity. Yet, when compared to other commonly used dementia detection tools developed for the primary care that have sensitivities as high as 0.54, the EDDA sensitivity of 0.57 is comparable.^55^ Furthermore, the study was conducted at a single health system, which may limit the generalizability of the findings to other health care settings with different patient populations or EHR systems. Finally, although the positive‐unlabeled learning approach enhanced model performance, it also introduced complexity in model interpretation, which may present challenges for clinical implementation. Future studies should include multicenter integration and validation of the EDDA, implementation and usability testing of the EDDA in real time, adapting the EDDA to predict future risk of dementia, adding more data sources to further enhance dementia detection (i.e., imaging and biomarker data), and integrating the algorithm into clinical care and assessing patient, care partner preferences, and impact on referrals and patient outcomes.

The EDDA demonstrates promising potential in enhancing dementia detection in the ED setting, addressing a critical gap in the timely identification of cognitive impairment among older adults. By leveraging machine learning models and positive‐unlabeled learning, EDDA can accurately alert clinicians to probable dementia at the point of care and identify dementia cases that might otherwise go unrecognized, thereby improving patient care and facilitating appropriate care transitions. Future work should focus on prospective validation across diverse health care systems, as well as integration into real‐time ED workflows to evaluate its impact on patient outcomes and health care utilization. Collaboration with clinicians and stakeholders will be essential to refine the model and ensure its successful adoption in clinical practice.

## CONFLICT OF INTEREST STATEMENT

Adam Mecca receives unrelated support through grants to Yale from the National Institutes of Health (NIH), Eli Lilly, Janssen, and Genentech. Andrew Taylor receives unrelated support from grants from the NIH, Gordon and Betty Moore Foundation, the Food and Drug Administration, the Agency for Healthcare Research & Quality (AHRQ), and Beckman Coulter, Inc., as well as options from Vera Health for serving as an advisor. Ula Hwang, Andrew Taylor, Inessa Cohen, Isaac Faustino, Natalia Festa, and Atharva Sapre are supported by the National Institute on Aging (NIA). All other authors declare that they have no known competing financial interests or personal relationships that could have appeared to influence the work reported in this paper. Author disclosures are available in the supporting information.

## Supporting information



Supporting Information

Supporting Information
