# Supplementary material for: Detection of emergency department patients at risk of dementia through artificial intelligence
Source: Alzheimers Dement. 2025 Jun 2;21(6):e70334. doi: 10.1002/alz.70334 (PMC12130574; doi:10.1002/alz.70334)
Supplement: Supplementary file 1 — Supporting Information [file ALZ-21-e70334-s001.docx]

Contents

[**Supplemental Figure 1. Predictors for Probable Dementia** 1](#_Toc190852762)

[**Appendix A. Modified Dementia Adjudication Protocol** 2](#_Toc190852763)

# **Supplemental Figure 1. Predictors for Probable Dementia**

A SHAP plot that shows the impact of various predictors on probable dementia in the validation and test sets. Each point represents an individual feature's SHAP value, with colors indicating the feature value (red for high, blue for low). Features to the left of the vertical line have a negative impact on the model's prediction (protective factors), while those to the right have a positive impact (risk factors).

Abbreviations: SHAP, SHapley Additive exPlanations.


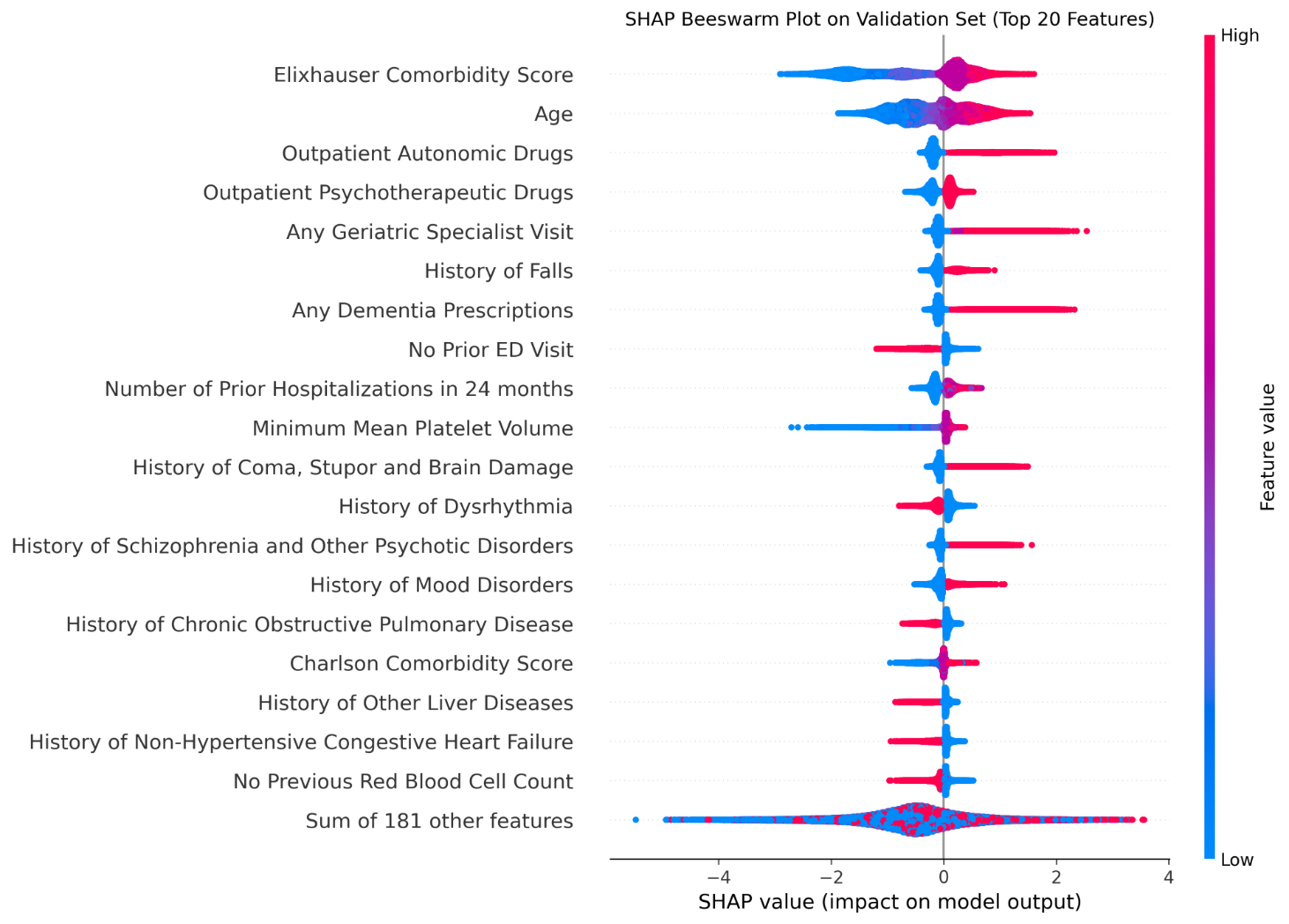


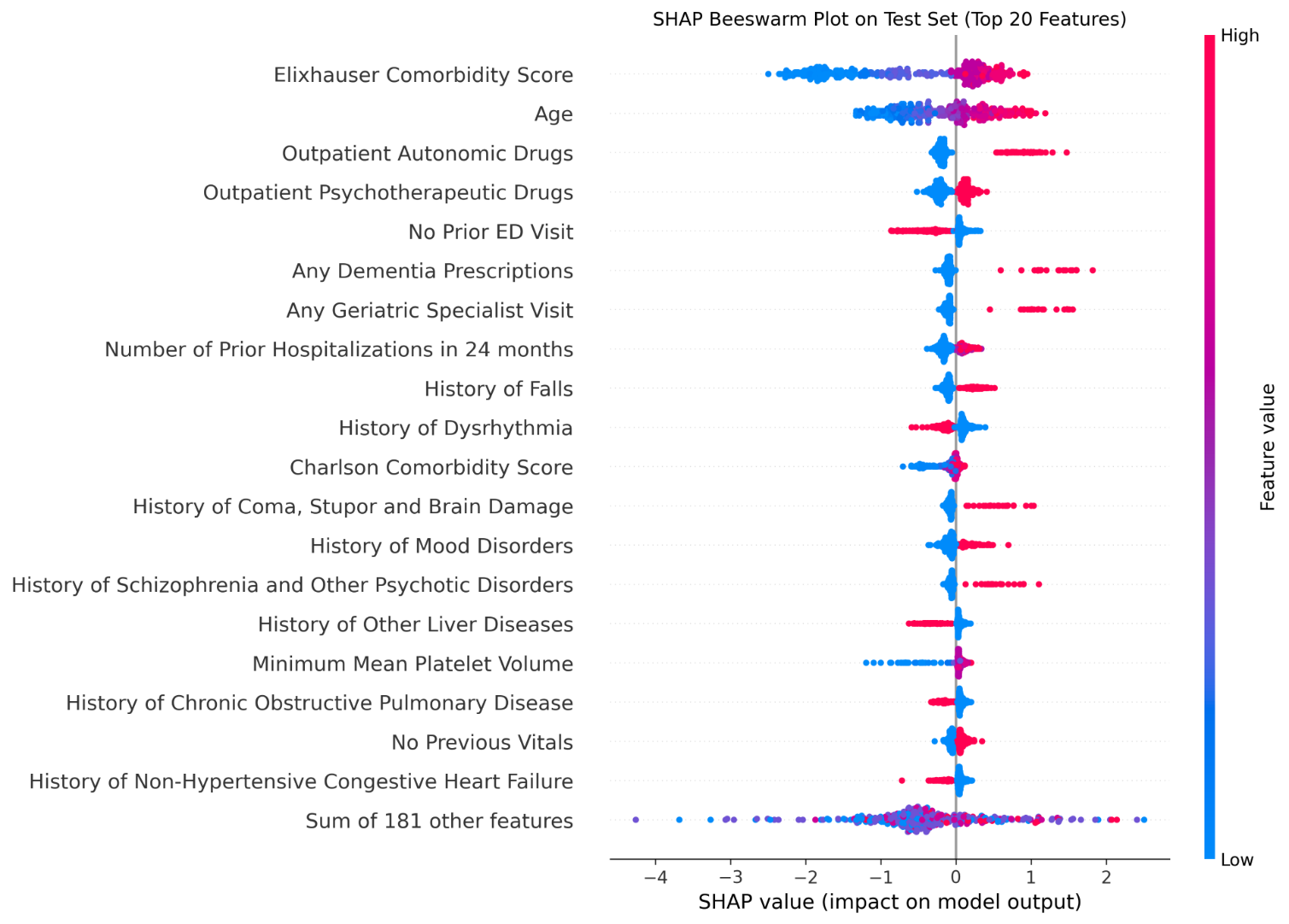


# **Appendix A. Modified Dementia Adjudication Protocol**

**Note**: This Standard Operating Procedure (SOP) duplicates some procedures and instructions from a previously published approach, to maintain consistency, while adapting the protocol to the current study. The original SOP is published within the supplementary materials of the following manuscript: Moura, L. M. V. R. *et al.* Identifying Medicare beneficiaries with dementia. *J. Am. Geriatr. Soc.* 69, 2240–2251 (2021).

**Overall Aim:** We aim to enhance access to appropriate diagnostics and healthcare for persons with dementia by improving identification among older adults with Emergency Department encounters from 01/2013 to 03/2022 in a large metropolitan health system. To do so, we developed a machine-learning instrument that classifies dementia status using available electronic health record and claims data. We plan to validate the performance of this instrument against a reference standard of clinician-adjudicated dementia, with a focus on discriminative power.

**Specific Aims:** Among patients 65-years and older with an emergency department encounter:

1. Accurately identify beneficiaries with true dementia using a modified version of a previously published adjudication procedure.
2. Estimate the performance characteristics of a novel instrument against a clinician-adjudicated reference standard for classification of dementia.

**Index:**

1. Demographic variables
2. Clinical variables for clinicians to extract from EHR [Epic] (i.e., “Clinical data” in REDCap”]
3. Tables
4. References
5. **DEMOGRAPHIC VARIABLES**
6. Age
7. Sex

**Unique Patient Identifier**

- - Number (unique patient ID number)

1. **CLINICAL VARIABLES FOR CLINICIANS TO EXTRACT FROM EHR**

**MINIMUM PROCESS:**

1. Set Time-frame: Review EHRs only between dates: 01/2013 to “**[Index Emergency Department Encounter]**.” Filter notes by date in Epic.
   1. Open patient’s EHR -> open “Chart Review” tab on left
   2. Open “Notes” tab in “Chart Review” ribbon
   3. Click “Filters” button in top left corner -> set “From:” date at “**01/2013**.” and “To:” date to “**[Index Emergency Department Encounter]**.”
   4. Click “Save as New Filter” and name it whatever you want -> This will automatically start filtering for this patient and will continue to use this filter until 1) you close out of Epic; 2) you manually uncheck this as a filter; 3) you manually hit “Clear Filters”, which is to the right of all available filters
   5. To use the same filter again after closing Epic: follow steps a and b -> then check the filter you named and saved from your prior session in the top filter ribbon.
   6. Alternatively, if you prefer to avoid filter functions, you can just sort notes by “Service Date” or “Specialty” once you open “Chart Review” -> Notes
2. First, review primarily (but not limited to) notes from primary care providers, psychiatry, psychology, social workers, neurology, neuropsychology, and geriatric medicine.
3. Second, review current medication list [ideally should consider medications used during the timeframe preceding the index Emergency Department Encounter.
4. Document cases with high diagnostic uncertainty for additional review and adjudication with the senior clinician
5. To access REDCap database: *TBD*
6. If a patient has no notes or encounters in Epic (i.e., missing data), then code “No data for abstraction” under first data variable. No other coding on REDCap is necessary beyond this point. If you desire, make a note under the NOTES variable to say data is missing in Epic. These cases were originally coded as “barely guessing” under cognitive concern.

**Clinician Adjudication Items:**

1. **Is there data available?**
   - 0- No data for abstraction (e.g., no notes, single encounter for care that does not offer insight into cognitive status (e.g., Podiatry care)
   - 1- Data for abstraction
2. **Is Mild Cognitive Impairment (MCI) or dementia present?**
   - 0- Cognitive concerns are not present
   - 1- Cognitive concerns are present
3. **Confidence in classification of cognitive concern**
   - 1 - Not at all confident
   - 2- Mildly confident
   - 3- Moderately confident
   - 4- Highly confident
4. **Stage classification (when cognitive concern is present)**
   - 1- MCI
   - 2- Dementia
   - 99- Q2 = 0

Notes: This is the reference-standard diagnosis for MCI and dementia. MCI and dementia criteria adapted from NIA-AA clinical criteria^1,2^ (Tables 2 and 3). The bedside testing required for Dementia criterion D might not be observed for many patients. For this adjudication, neuropsychology testing is not required if there is adequate description of functional and cognitive decline.

1. **Confidence in classification of stage**
   - 1 - Not at all confident
   - 2- Mildly confident
   - 3- Moderately confident
   - 4- Highly confident
   - 99- Q2 = 0 or Q4 = 1
2. **Severity (when dementia is present)**
   - 1- Mild
   - 2- Moderate
   - 3- Severe
   - 9- Unknown
3. **Confidence in classification of severity**
   - 1 - Not at all confident
   - 2- Mildly confident
   - 3- Moderately confident
   - 4- Highly confident
   - 99- Q2 = 0 or Q4 = 1 or 99

Notes: Severity staging, which is detailed in Table 4, is adapted from DSM-5 diagnosis criteria for major neurocognitive impairment^3^. NIA-AA dementia diagnostic criteria^2^ does not address severity.

**8. Concern for Substance Use Past or Present?**

- 0 – No
- 1 – Yes

**9. Was Q4 = 2? (Automated column)**

- If Q2 = 2 (Dementia), patient was marked as having dementia
- If Q2 = 1 (MCI) or Q2 = 99 (N/A, ie Q2 = 0), patient was marked as not having dementia
- If Q2 is blank, adjudicator needs to revisit Q2
